# The Emerging Roles of Human Leukocyte Antigen-F in Immune Modulation and Viral Infection

**DOI:** 10.3389/fimmu.2019.00964

**Published:** 2019-05-10

**Authors:** Aifen Lin, Wei-Hua Yan

**Affiliations:** ^1^Biological Resource Center, Taizhou Hospital of Zhejiang Province, Wenzhou Medical University, Linhai, China; ^2^Medical Research Center, Taizhou Hospital of Zhejiang Province, Wenzhou Medical University, Linhai, China

**Keywords:** human leukocyte antigen F, immune cells, receptor, immune regulation, viral infection

## Abstract

Human leukocyte antigens (HLAs) play various critical roles in both innate and adaptive immunity through processes such as presenting antigens to T cells and serving as ligands for receptors expressed on natural killer (NK) cells. Among the HLA class I family, the clinical significance and biological function of HLA-F have been the least investigated and have remained elusive for a long period of time. Previous studies have revealed that HLA-F expression might be involved in various physiological and pathological processes, such as pregnancy, viral infection, cancer, transplantation, and autoimmune diseases. However, recent data have shown that, akin to other HLA family members, HLA-F molecules can interact with both activating and inhibitory receptors on immune cells, such as NK cells, and can present a diverse panel of peptides. These important findings pave new avenues for investigations regarding the functions of HLA-F as an important immune regulatory molecule. In the present review, we summarize the studies on the role of HLA-F in immune modulation, with a special emphasis placed on the roles of HLA-F and KIR3DS1 interactions in viral infection.

## Introduction

Human leukocyte antigen (HLA) class I antigens have multiple important functions in immune regulation. The functions of HLA class I antigens are involved in presenting antigens to T cells and serving as ligands for a panel of immune receptors expressed on natural killer (NK) cells, T cells, and myeloid cells (1, 2). The HLA class I family can be further grouped as the classical (HLA-Ia) and nonclassical antigens (HLA-Ib). The HLA-Ia group includes the HLA-A, -B, and -C molecules, and the HLA-Ib group comprises the HLA-E, -F, and -G molecules (3). In contrast to the highly polymorphic HLA-Ia antigens that are ubiquitously expressed on all nucleated cells, HLA-Ib molecules are characterized by particular tissue localizations, low genetic diversity, a limited peptide repertoire, and functional profile (4).

HLA-E, HLA-F, and HLA-G were first identified at almost the same time during the late 1980s (5–7), and the biological function and clinical significance of HLA-G and HLA-E have been more intensively investigated than those of HLA-F (3). During the past several decades, in addition to its initial discovery on extravillous cytotrophoblasts, the aberrant expression of HLA-G has been observed in many disorders (8). The immune suppressive potential of HLA-G has also been established, and HLA-G can obviously inhibit the functions of immune cells, such as natural killer (NK) cells (9), T cells (10), dendritic cells (DCs) (11), neutrophils, and B cells, through the immune inhibitory receptors immunoglobulin (Ig)-like transcript receptor 2 (ILT2) and/or ILT4, and the HLA-G/ILT signaling pathway, acting as an immune checkpoint, has also been recognized (12–14). HLA-E can affect both innate and adaptive immune responses by interacting with the inhibitory CD94-NKG2A receptor and also with the activating receptor NKG2C on NK cells and the CD8 receptor on T cells (15).

Since the discovery of HLA-F (HLA-5.4) in 1990 by Geraghty and coworkers, only a small amount of evidence for its clinical relevance has been obtained (6, 16). For example, the genetic variants and protein expression of HLA-F have been observed to be associated with different types of diseases, such as cancer (17–19), infection (20, 21), reproduction, and autoimmune disorders (22–25). Elevated HLA-F expression has been found in cancer lesions and peripheral blood, which was associated with poor survival in cancer patients (19, 26). More recently, HLA-F expression was found to be upregulated and to, perhaps, play an important role during the progression of viral infection (21, 27). However, the underlying mechanism of HLA-F activity has remained enigmatic for decades, and only recently has it become clear that it is an important immune regulator. Recent data revealed that, akin to other HLA family members, HLA-F molecules can interact with both activating and inhibitory receptors on immune cells such as NK cells and can present a diverse panel of peptides to T cells (28–30). In this scenario, the open conformers (OCs) of HLA-F molecules have been observed to directly bind to immune inhibitory receptors (KIR3DL2 and ILT2) and immune-activating receptors (KIR2DS4 and KIR3DS1) on NK cells (27, 29, 31); additionally, HLA-F has been shown to present peptides to T cells and to regulate immunity through interactions with distinct NK cell receptors, depending on the molecular conformation of peptide-bound HLA-F or HLA-F OCs (30). These important findings provide new evidence that HLA-F functions as an important immune regulatory molecule, and evidence for the significance of HLA-F in human physiological and pathological conditions has been emerging (31–33).

In the present review, we will focus on the potential roles of HLA-F in immune modulation and its relevance in infectious disorders.

## Molecular Structure and Expression of Human Leukocycte Antigen-F

Limited genetic variation is one of the most obvious features of the HLA-Ib family. To date, there are only 31 alleles for *HLA-F* at the DNA level, which can encode six distinct HLA-F proteins (HLA-F^*^01:01, HLA-F^*^01:02, HLA-F^*^01:03, HLA-F^*^01:04, HLA-F^*^01:05, and HLA-F^*^01:06; http://www.anthonynolan.org/HIG/, and another nine novel *HLA-F* alleles have been discovered that will be added to the database (34). Among these *HLA-F* alleles, HLA-F^*^01:01 has the highest frequency, followed by HLA-F^*^01:03, in different populations globally (35).

The structure of the *HLA-F* gene is similar to that of the other *HLA-I* genes, which consist of eight exons; however, the 3' untranslated region of *HLA-F* is different from those of the other *HLA-I* genes (6). Among these exons, exon 1 generates the signal peptide; exons 2–4 generate the HLA-F extracellular domains, α1, α2, and α3; exon 5 generates the transmembrane domain; and exon 6 generates the intracellular cytoplasmic tail of the HLA-F protein. Because exon 7 remains untranslated, the cytoplasmic tail of HLA-F is much shorter (~2 kDa) than those of the other HLA-I molecules. Moreover, the length of the cytoplasmic tail of HLA-F varies, which can lead to the generation of different HLA-F isoforms (36).

HLA-F expression has been found primarily within cells. In this scenario, intracellular HLA-F expression was frequently observed in resting cells, such as peripheral blood B cells, B cell lines, and tissues, such as the fetal liver, adult tonsils, and thymus, and in malignant lesions or tumor cell lines (37). However, cell surface HLA-F expression has been detected on activated B cells, T cells, NK cells, Epstein-Barr virus (EBV)-transformed B lymphoblastoid cell lines (38), virus-infected cells (21, 27), and on other tissues such as insulin-containing islets (39) and first- and second-trimester migratory and invasive extravillous cytotrophoblasts (22, 23). HLA-F messenger RNA (mRNA) transcription and cell surface expression can be induced on activated CD4+ T cells stimulated by the cytokine interleukin-2 (IL-2) and by the biochemicals phorbol 12-myristate 13-acetate and ionomycin (27). Unlike most HLA-I antigens, the stability and transportation of HLA-F from the endoplasmic reticulum (ER) have been found to be independent of tapasin and transporter associated with antigen processing (TAP), which play critical roles in appropriate peptide loading (40). However, HLA-F export from the ER is entirely dependent on a C-terminal valine residue and Golgi localization-related RxR motifs in its cytoplasmic tail (41).

Cell surface HLA-F can be expressed as a heterotrimer complex of the HLA-F heavy chain with β_2_m and peptides, and only the HLA-F heavy chain can be expressed as an open conformer (OC) without peptides or β_2_m (27, 30, 31). Among the HLA-I OCs, HLA-F OCs seem to be more stable, and HLA-F OCs can bind different allelic HLA-I OCs and cooperate in the uptake of extracellular antigens for cross-presentation (31, 42). The distinct conformation of cell surface HLA-F molecules can dramatically affect their biological functions due to differential binding to either activating or inhibitory natural killer cell receptors (NKRs) expressed on various immune cells, such as HLA-F OCs can bind to the inhibitory receptor killer Ig-like receptor (KIR) 3DL2 and the activating receptor KIR3DS1 (27, 31). However, unlike the HLA-F OCs, the HLA-F:β_2_m:peptide complex can bind to the inhibitory receptors ILT2 [leukocyte Ig-like receptors (LILR)-B1] and ILT-4 (LILR-B2), which is based on a highly conserved docking orientation on the side of the HLA-F:β_2_m:peptide complex (30).

Furthermore, Dulberger et al. (30) also showed that HLA-F can present unique unconventional long peptides (ranging from 7 to 30+ residues and peaking at 12 residues) through an open-ended groove as a result of an R62W substitution in the HLA-F heavy chain. The R62W substitution blocks the N-terminal anchor pocket of HLA-F. Consequently, HLA-F anchors the peptides at its C terminus. Distinct from the peptides (typically 8–10 residues) presented by other HLA-I molecules, the extra-long peptides presented by HLA-F were much more similar to those of HLA-II molecules (30). However, whether there are HLA-F antigen-specific recognition by T cell receptors (TCRs) or NKRs remains to be explored.

## Immune Receptors for Human Leukocyte Antigen-F

**Figure 1:**
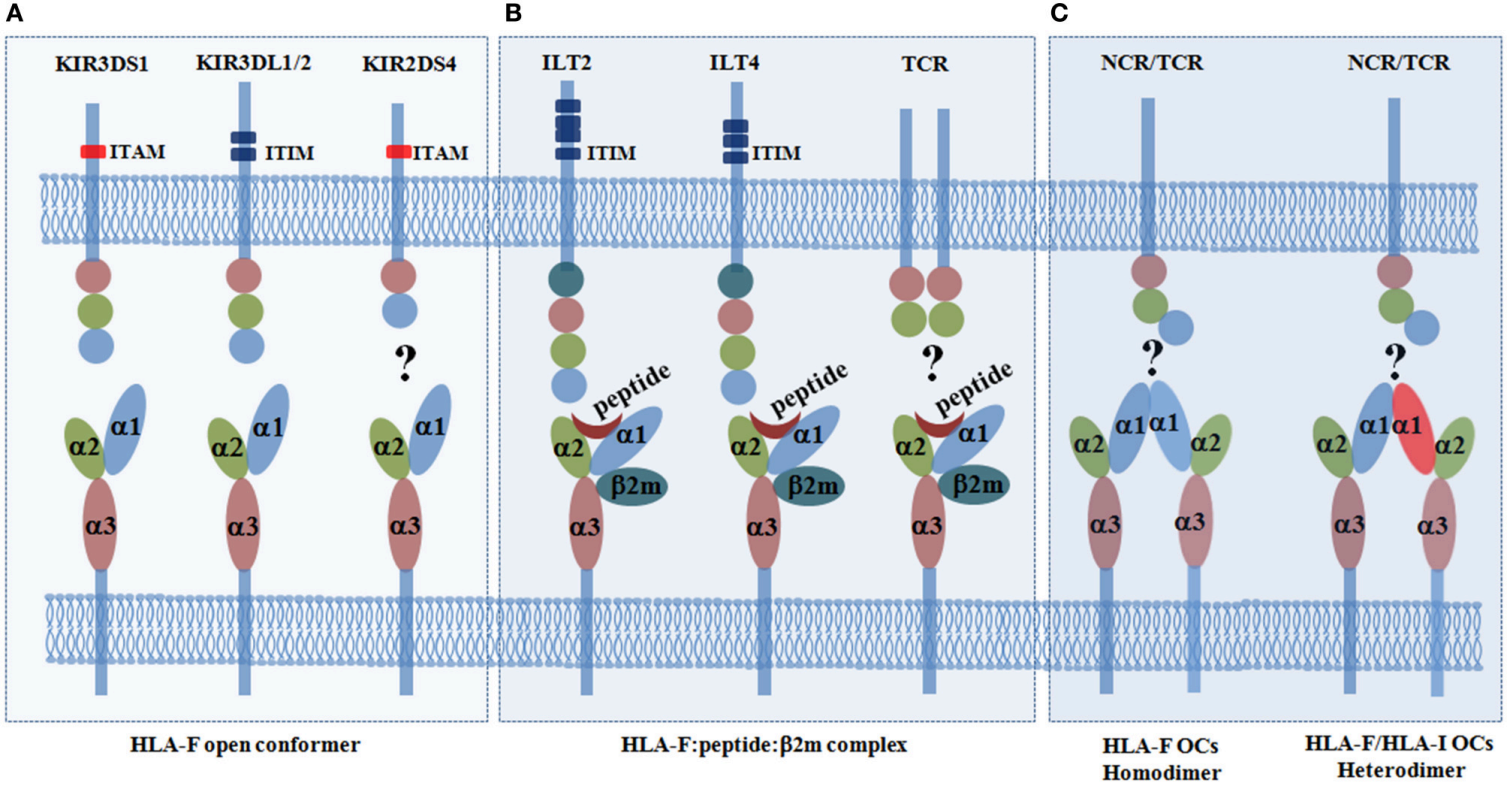
Immune receptors for the human leukocyte antigen (HLA-F) open conformer (OC) and HLA-F:peptide:β_2_m complex. **(A)** HLA-F OCs can be recognized with the highest affinity by the activating receptor KIR3DS1 and can be recognized by the inhibitory receptors KIR3DL1/2. However, the results regarding the interaction of the HLA-F OC and the activating receptor KIR2DS4 are disputed (27, 28, 30, 31). **(B)** The HLA-F:peptide:β_2_m complex can only be recognized by the inhibitory receptors immunoglobulin (Ig)-like transcript receptor 2 (ILT2) and ILT4 (29, 30). However, whether there are HLA-F peptide-restricted T cell receptors (TCRs) is not yet known (30). **(C)** HLA-I OCs can be expressed on the cell surface as homodimers (43), and it has been postulated that HLA-I/HLA-F heterodimers can form (44). Whether HLA-F can be recognized by other TCRs or natural killer cell receptors (NCRs) remains to be explored. Killer cell immunoglobulin-like receptors (KIRs) are transmembrane glycoproteins expressed on natural killer (NK) cells and on subpopulations of T cells. KIRs have either two (2D) or three (3D) extracellular immunoglobulin (Ig)-like domains and different cytoplasmic tails (short or long). Inhibitory KIRs have long cytoplasmic tails (L) that contain immunoreceptor tyrosine-based inhibition motifs (ITIMs). Activating KIRs have short cytoplasmic tails (S) that lack ITIMs but associate with the immunoreceptor tyrosine-based activation motif (ITAM), such as the adapter DAP12, *via* a positively charged arginine or lysine residue in their transmembrane domain. Immunoglobulin-like transcript 2 (ILT2, also known as CD85j and LIR1) has four extracellular Ig-like domains and four cytoplasmic ITIMs. ILT4 (also known as CD85d and LIR2) has four extracellular Ig-like domains and three cytoplasmic ITIMs. TCRs (T cell receptors) recognize peptides presented by HLA molecules.

The immune receptors that can bind to HLA-F had remained elusive until recent discoveries. During the past several decades, several receptors among the super families of LILRs (ILT2 and ILT4) and KIRs (3DL1, 3DL2, 3DS1, and 2DS4) have been found to bind HLA-F, although the findings on HLA-F–KIR2DS4 binding are controversial (33). The details of the HLA-F and ligand interaction are presented in Figures 1A,B. LILRs are in close linkage with the KIR family and are encoded by the genes located in the leukocyte receptor cluster on chromosome 19q13.4, and both of these families share similar immunoglobulin (Ig)-like structure and cytoplasmic signaling domains (45). KIRs are predominately expressed on NK cells but are also expressed on subpopulations of CD4+ T cells, CD8+ αβ T cells, and γδ T cells (46). ILTs are broadly expressed on NK cells and T cells, as well as on DCs, B cells, macrophages, and monocytes (47). Functionally, ILT2, ILT4, and KIR3DL2 are immune inhibitory receptors that are characterized by an immunoreceptor tyrosine-based inhibition motif (ITIM) in their long intracytoplasmic tails. KIR3DS1 and KIR2DS4 are immune-activating receptors that are characterized by short intracytoplasmic tails that are associated with a transmembrane immunoreceptor tyrosine-based activation motif (ITAM), such as DAP12 (48, 49).

Garcia-Beltran et al. (27) systematically analyzed the KIR–HLA interactions. In their study, the binding capability of the KIR-Fc fusion protein was screened by 100 HLA-I allotypes in two conformational states with both HLA-I complexes (untreated) and HLA-I OCs (acid pulsed). Using surface plasmon resonance, KIR3DS1ζ^+^ Jurkat reporter cell assays, and KIR3DS1^+^ NK cell function evaluation, they reported for the first time that KIR3DS1 can bind HLA-F OCs with high affinity and with a relatively small dissociation rate. Moreover, the study revealed that KIR3DL1 and KIR3DL2 can also bind HLA-F OCs with less affinity than KIR3DS1, but no binding was observed between KIR2DS4 and HLA-F OCs. Shortly thereafter, a study by Burian et al. (31) reinforced the findings that the binding of KIR3DS1 to HLA-F OCs is higher than that of most of the HLA-I allotypes tested, and the affinity of KIR3DS1–HLA-F OCs was found to be eightfold higher than that of the KIR3DL1–HLA-F OC interaction. However, the authors found that KIR2DS4 can bind to HLA-F OCs in pull-down experiments, which is in agreement with a previous study that revealed that KIR2DS4, as well as KIR3DL2, can be KIR receptors for the recognition of HLA-F OCs (28).

Unlike KIRs that bind HLA-F OCs, the inhibitory receptors ILT2 and ILT4 have been reported to recognize the complex of HLA-F heavy chain with β_2_m and peptide (29). In addition to the HLA-F complex, ILT2 and ILT4 also recognize other HLA-Ia and HLA-Ib molecules, such as HLA-G (50). ILT2 binds to the α3 domain of the HLA-I heavy chain and β_2_m. However, ILT4 can also recognize free HLA-I heavy chains (51). The crystal structure of the HLA-F:β_2_m:peptide complex was solved recently, which finally unveiled the molecular structure of HLA-F and its receptor binding profile. Taking advantage of this molecular structure, details of the docking mode of ILT2 interacting with the HLA-F:β_2_m:peptide complex were determined. Biolayer interferometry measurements showed that ILT2 does recognize the HLA-F:β_2_m:peptide complex but not HLA-F OCs, with the highest affinity of 2 μM among the HLA-I molecules (30). The crystal structure revealed that ILT2 adopts a highly conserved docking orientation, recognizing one side of the β_2_m and α3 domain of the HLA-F complex, which is away from the peptide-presenting groove. The data also explained why KIRs, such as KIR3DS1 and KIR3DL2, can only recognize HLA-F OCs but not peptide-bound HLA-F and that the binding of peptides can directly hinder the KIR–HLA-F:β_2_m:peptide complex interaction. Furthermore, HLA-I OCs can be expressed on the cell surface as homodimers (43), and it is postulated that they may be expressed as HLA-I/HLA-F heterodimers, which could cooperate with other HLA-I OCs in exogenous antigen cross-presentation (44). Because HLA-F has an open-ended antigen-binding groove that can bind a diverse number of uncharacteristically long peptides and it can cooperate with other HLA-I OCs, whether HLA-F can be recognized by other TCRs or KIRs also should be explored (Figure 1C). Indeed, HLA-B^*^35:01 OCs preferentially bound CD8+ T cells through a CD8-dependent binding mode, but the HLA protein did not interact with the TCR (52). This kind of binding is different from that of TCR and the coreceptor CD8, which can simultaneously recognize a specific peptide–major histocompatibility complex (MHC)-I complex on the surface of antigen-presenting cells and induce CD8+ T cell activation.

## Function of Human Leukocyte Antigen-F and KIR3DS1 Interaction in Viral Infection

During viral infection, disease progression and outcome depend on host immune responses and viral factor interactions. Both innate and adaptive immune cells play critical roles in counteracting viral infection, although viruses have developed various strategies to escape host antiviral immune responses (53). One key mechanism that viruses use to shape host immune recognition and attack is the alteration of HLA antigen expression on infected cells (54, 55). Fortunately, a large body of studies have revealed that particular KIR and HLA molecule interactions are significant in viral infection protection and control (56, 57).

For example, previous studies have demonstrated the association of certain KIR3DL1 allotypes and the KIR3DS1–HLA-Bw4^**80I**^ (HLA-Bw4 molecules have an isoleucine in position 80) interaction with various types of viral infection, although the evidence for the direct binding between KIR3DS1 and HLA-Bw4 remains inconclusive (27, 58–61). In previous studies, several potential ligands for KIR3DS1 have been proposed. Li and coauthors (62) found that HLA-B^*^2705 can directly bind KIR3DS1 with an affinity of 6.95 × 10^−6^ mol/L. A study by O'Connor et al. (63) showed that KIR3DS1 can recognize HLA-B^*^57:01 in a specific peptide-dependent manner. Additionally, HLA-B^*^51 has been reported to be recognized by KIR3DS1 (64). KIR3DS1 is an immune-activating receptor that associates with the ITAM-bearing adaptor DAP12, which can induce NK cell cytolysis and Interferon (IFN)-γ production, such as with KIR3DS1-specific antibody linking (49). Recently, it was uncovered that KIR3DS1 binds HLA-F OCs. This finding greatly contributes to a deeper understanding of the immune regulation and clinical significance of HLA-F through KIR3DS1 recognition. Indeed, the importance of specific HLA-F-KIR3DS1 binding and its function in infectious disease is emerging.

A study by Kumar et al. (65) revealed that intracellular HLA-F protein expression can be induced by Japanese encephalitis virus in human amniotic and brain microendothelial cell lines, which is dependent on the DNA-binding protein Nuclear factor-κB (NF-κB). Using KIR3DS1^+^ and KIR3DS1^−^ NK cell clones (NKCLs), Garcia-Beltran et al. (27) found that HLA-F OCs can only trigger the immune functions of KIR3DS1^+^ NK cells, such as degranulation and IFN-γ, Tumor Necrosis Factor (TNF)-α, and chemokine (CC-motif) ligand 4 (CCL4) cytokine/chemokine production, and that only KIR3DS1+ NK cells can effectively inhibit HIV replication in HIV-infected autologous CD4+ T cells. Although HIV infection can induce HLA-F mRNA transcription in CD4– or CD4+ T cells, cell surface HLA-F expression has been observed to be decreased with HIV infection in T cells. These results indicate that HIV infection can impair cell surface HLA-F expression, which may favor viral immune evasion; however, it seems that the downregulation of HLA-F expression is not sufficient to counteract the killing effects mediated by the HLA-F–KIR3DS1 interaction. KIR3DS1+ NK cells have been observed to be stimulated by HLA-F expressed on HLA-null 721.221 cells with the induction of CCL4, IFN-γ production, and CD107a expression (66). Activated NK cells secrete cytokines, chemokines, and degranulate have been well-documented as limiting viral infection, including the chemokine CCL4, which can inhibit HIV replication and entry into target cells (66, 67).

With multiple models, such as *in vitro* cell culture and *in vivo* chimeric humanized mouse models and clinical tissues, the observation that hepatitis C virus (HCV) infection induces HLA-F expression has been well-established by Lunemann and coworkers (21). These researchers found that HCV infection can significantly upregulate cell surface HLA-F expression on the hepatoma cell line Huh7.5, which lacks intrinsic HLA-F expression. Moreover, HCV-induced HLA-F expression existed only in the livers of HCV-infected humanized chimeric mice and was more frequently observed in liver biopsies from HCV-infected patients. The cell surface HLA-F expression was further confirmed by a binding assay and activation assay with the KIR3DS1-Fc fusion protein and the KIR3DS1ζ-Jurkat reporter cell line, respectively. As well as inhibiting HIV replication, KIR3DS1+ NK cells have potent antiviral capabilities during HCV infection.

## Conclusion

HLA molecules can be specifically recognized by different types of TCRs and NKRs, which are vital to maintain homeostasis or elicit an immune response to attack foreign or infectious antigens (68). Very recently, the importance of HLA-F in immune regulation has been uncovered, suggesting that HLA-F can present a diverse array of uncharacteristically long peptides, and HLA-F associated with a β_2_m and peptide complex and HLA-F OCs can be recognized by distinct NKRs. In this context, ILT2 and ILT4 bind HLA-F associated with a β_2_m and peptide complex through a docking strategy that precludes HLA-F OC recognition. However, KIRs (3DL1, 3DL2, 3DS1, and 2DS4) bind HLA-F OCs, and HLA-F OCs have the highest binding affinity for KIR3DS1 among these KIRs (27, 30).

In viral infection, HLA-F acts as a stress signal on virus-infected cells, which activates KIR3DS1+ NK cells, inhibiting viral replication and killing the infected cells. In line with this observation, the HLA-F OC and KIR3DS1 interaction elicits antiviral immune responses, such as increased NK cell cytolysis and cytokine production, which inhibit HIV-1 and HCV replication, suggesting a mechanism for KIR3DS1 in viral infection protection and viral clearance (21, 54, 69). However, many key questions remain to be answered. As KIR3DS1 is also expressed on subsets of T cells that may have effects on the regulation of T cell function, a potential role of HLA-F in KIR3DS1+ T cells remains to be explored (70). Moreover, little evidence is currently available on the functions of HLA-F with other receptors, including the inhibitory receptors ILT2, ILT4, KIR3DL1, and KIR3DL2, and the activating receptor 2DS4; their interaction and whether they are implicated in diseases have to be investigated. As previous studies revealed that an increased tumor lesion HLA-F expression has been associated with poor prognosis in patients with gliomas (71), nasopharyngeal carcinoma (26), stage II breast cancer (72), and esophageal squamous cell carcinoma (19). These findings indicated that HLA-F expression might contribute to cancer cells escaping from immune surveillance and antitumor immune responses through the binding to the inhibitory receptors such as ILT2 and ILT4 expressed on immune cells. Indeed, ILT2 and ILT4 expression has been observed in tumor-infiltrating immune cells such as CD4+ and CD8+ T cells (73). However, interaction and signal transduction between the receptors and the tumor cell expressed HLA-F antigens remain to be explored. Finally, as an antigen presenter, future studies for peptide sequence-restricted HLA-F T cells or NK cell receptors are needed. Indeed, data obtained from HLA-F tetramers and peripheral blood mononuclear cell binding assays revealed that the peptide presented by HLA-F has a dramatic effect on the staining profile, making it possible that there are receptors that can differentiate the HLA-F complex bound to different peptide sequences (30).

In summary, with the discovery of its ability to bind NKRs, particularly KIR3DS1, which are involved in various physiological and pathological processes, the clinical significance of HLA-F is emerging.

## Author Contributions

All authors listed have made a substantial, direct and intellectual contribution to the work, and approved it for publication.

### Conflict of Interest Statement

The authors declare that the research was conducted in the absence of any commercial or financial relationships that could be construed as a potential conflict of interest.
